# Sesamol ameliorates hypotension by modulating cytokines and PPAR-gamma in systemic inflammatory response

**DOI:** 10.17179/excli2015-367

**Published:** 2015-08-13

**Authors:** Srinivasan Periasamy, Pei-Yi Chu, Ya-Hui Li, Dur-Zong Hsu, Ming-Yie Liu

**Affiliations:** 1 Department of Environmental and Occupational Health, College of Medicine, National Cheng Kung University, Tainan, Taiwan

**Keywords:** acute renal injury, hypotension, inflammatory cytokines, proliferator-activated receptor-gamma, lipopolysaccharides, sesamol, sepsis

## Abstract

Sepsis is one of the major causes of death reported in intensive care units. Acute kidney injury (AKI) and hypotension are important in the pathogenesis and mortality of systemic inflammatory response (SIR). Sesamol delays mortality in sepsis; however, its effects on AKI and hypotension and the role of peroxisome proliferator-activated receptor-ɣ (PPAR-γ) activation have not been established. We investigated the effect of sesamol on SIR in cecal ligation and puncture (CLP)-induced acute kidney injury and lipopolysaccharide (LPS)-induced hypotension in rats. Sesamol was subcutaneously injected 1 h after SIR. Renal function (BUN and CRE) and proinflammatory mediators interleukin (IL)-1β and IL-6 were increased after CLP. Tumor necrosis factor (TNF)-α, IL-1β, IL-10, and nitrite production were significantly increased 6 h after LPS-induced hypotension (mean arterial pressure was significantly decreased). Sesamol significantly inhibited BUN, CRE, IL-1β, IL-6, and nitrite after CLP-induced acute renal injury. In addition, sesamol increased mean arterial pressure and IL-10, inhibited TNF-α and IL-1β, but did not affect nitrite production in LPS-induced hypotension. Sesamol increased PPAR-γ in the leucocytes and peritoneal macrophages in LPS-induced SIR. We conclude that sesamol regulates leucocyte and macrophage PPAR-γ-associated systemic cytokines expression, thereby ameliorates acute kidney injury and hypotension in rats.

## Introduction

Sepsis ranks among the top ten causes of mortality in the USA intensive care units (Angus et al., 2001[1]). Infection mediated sepsis is closely associated with systemic inflammatory response (SIR) (Oshima et al., 2005[24]). Systemic inflammation is strongly associated with acute kidney injury. During sepsis massive production of proinflammatory cytokines that leads to hypotension culminating in multiple organ failure. The most common cause of mortality is acute kidney injury (AKI). AKI is strongly associated with systemic inflammation (Yegenaga et al., 2004[34]). Cecal ligation and puncture (CLP) is a familiar rodent model that produces polymicrobial infection with systemic inflammation. Polymicrobial infection elicits the discharge of proinflammatory cytokines in blood and induces AKI (Yasuda et al., 2006[33]). CLP caused a pronounced fall in mean arterial pressure and renal dysfunction, as measured by plasma creatinine and urea levels (Doi et al., 2008[10]). However, measuring blood pressure (mean arterial pressure) in CLP-induced animal is impossible, as the blood pressure falls beyond the instruments measuring level and using anesthesia also interferes in measuring blood pressure and results in mortality. Therefore, we used lipopolysaccharide (LPS)-induced SIR for further studies.

LPS, the main component of the outer cell wall in all gram-negative bacteria triggers severe pathological alterations. Endotoxemia is associated with systemic inflammation, hypotension, cardiac dysfunction, altered oxygen consumption, multiple organ failure, and death in the clinical study (Murray, 1998[23]). Endotoxemic experimental animals are characterized by the increase in proinflammatory cytokines, leading to development of systemic hypotension (Liu et al., 1997[20]). Inflammatory cytokines and nitric oxide are responsible for activating and aggravating SIR, both of which result in the septic hypotension (Wort and Evans, 1999[31]; Zhang, 2008[35]). Pro-inflammatory mediators including tumor necrosis factor (TNF)-α, interleukin (IL)-1β, and nitric oxide are involved in the pathogenesis of septic hypotension (Dinarello, 1997[9]). Anti-inflammatory cytokine IL-10 is important in resolving inflammatory response (Walley et al., 1996[30]). Inflammatory cells, including antigen presenting cells, monocytes, and macrophages, are found to possess peroxisome proliferator-activated receptor (PPAR)-γ (Miksa et al., 2007[22]). Activated PPAR-γ inhibits the expression of pro-inflammatory mediators by inflammatory cells (Chinetti et al., 2000[4]).

Sesamol (3,4-methylenedioxyphenol), an active component of sesame oil, derived from the plant species *Sesamum indicum *L., attenuates lipid peroxidation to protect against multiple organ injury (Hsu and Liu, 2004[15]). Sesamol is an effective nonfat, water soluble, antioxidant lignan with potent anti-oxidative and anti-inflammatory effects. Sesamol decreases oxidative stress, inflammation, multiple organ damages, and mortality in septic rats (Hsu et al., 2006[12]; Chu et al., 2010[5]). However, the effect of sesamol on SIR induced AKI and hypotension has never been investigated. Thus, we investigated the effect of sesamol on SIR-induced AKI and hypotension and the role of PPAR-γ in rats.

## Materials and Methods

### Materials 

Sesamol and LPS were purchased from Sigma-Aldrich (St Louis, MO). All other chemicals obtained were research grade.

### Animals

Male Wistar rats (200-300 g) were obtained from and housed in our institution's Laboratory Animal Center. Rats were housed in polypropylene cages (3 rats/cage) in a room with 12 h light/dark cycle and with central air conditioning (25 C, 70 % humidity), throughout the experimental period. They were given pellet feed (Richmond Standard; PMI Feeds, Inc., St. Louis, MO) and water *ad libitum*. The experimental protocols and animal care were in agreement with nationally permitted guidelines.

### Experimental design

#### CLP-induced systemic inflammation

The anterior abdominal wall of light diethylether anesthetized rats was shaved. To expose the cecum and the adjacent intestine a 2 cm midline opening were made. The cecum was ligated and perforated twice with an 18-gauge needle; the cecum was gently pressed to ooze fecal material. The abdominal opening was sutured, and 1 mL of saline was injected (s.c.) for fluid restoration. 

#### Design

Four groups rats each (n = 5). Group I: sham operation without CLP. Group II: CLP only. Group III: sesamol (s.c.) (10 mg/kg) 0 and 6 h after CLP. Group IV: rats were given subcutaneous (s.c.) sesamol same as Group III. Serum blood urea nitrogen (BUN) and creatinine (CRE) levels, and IL-1β and IL-6 levels were determined 12 h after CLP.

### LPS induced systemic inflammation

#### LPS-induced hypotension

Rats were anesthetized with sodium pentobarbital (50 mg/kg, (intraperitoneal) i.p.). LPS (10 mg/kg, (intravenous) i.v.) was injected to induce hypotension. LPS derived from *Escherichia coli* serotype O55:B5, and sesamol were obtained from Sigma-Aldrich (St. Louis, MO).

#### Study A

Rats were divided into three groups of six. LPS group, rats were treated with LPS (10 mg/kg, i.v.). LSM1- and LSM3-group rats were treated with sesamol (1 and 3 mg/ kg, s.c., respectively) 1 h after LPS injection. Mean arterial pressure (MAP) was measured at 0, 1, 3, and 6 h after LPS injection.

#### Study B

Rats were divided into four groups: N group, rats were given saline only; SM group, rats were given sesamol (3 mg/kg, s.c.); LPS group, rats were given LPS (10 mg/kg, i.v.); LSM group, rats were given LPS then sesamol 1 h after LPS. TNF-α, IL-1β, IL-10, and nitrite levels in serum (n = 6) and transcription factor PPAR-γ activation in leukocytes (n = 4) were measured 6 h after LPS injection.

#### Study C

Peritoneal macrophages were subjected to one of five treatments: Group I, cells were treated with PBS only; Group II, cells were treated with LPS (100 ng/mL); Group III-V, cells were treated with LPS (100 ng/mL) plus sesamol (3 νΝ, 30 νΝ, or 300 νM). The level of PPAR-γ activation and cell viability were measured 24 h after LPS treatment.

### Blood collection

Under light ethylether anesthesia blood samples were collected from the femoral vein. Serum separation tubes were used for blood collection by venipuncture and let to clot at room temperature for 30 min, centrifuged at 2500 rpm for 10 min at 4 C. 

### Assessing kidney dysfunction 

Biochemical analyzer (DRI-CHEM 3500 s; Fujifilm, Kanagawa, Japan) were used to evaluate BUN and CRE in blood. Kidney dysfunction was evaluated by quantifying increases in serum levels of BUN and CRE. 

### Measuring blood pressure

Rats were anesthetized with sodium pentobarbital (50 mg/kg, i.p.). The femoral arterial cannula was connected to a volumetric pressure transducer (Diagnostic & Research Instruments Co, Taipei, Taiwan). MAP measurements were done on individual (same) animals during the whole experimental period of 6 h. MAP was monitored and recorded on a polygraph (Diagnostic & Research Instruments Co, Taipei, Taiwan). XctionView Data Acquisition software was used to analyze the data.

### Measuring cytokines in serum 

The TNF-α, IL-1β, IL-10, and IL-6 levels in serum were evalauted quantitatively using enzyme-linked immunosorbent assay (ELISA) kits (DuoSet; R&D Systems Inc., Minneapolis, MN, USA) (Chu et al., 2010[6]). 

### Measuring nitrite in serum

The nitrite content in the serum was measured by Griess reaction. Incubating 100 νL of serum with 100 νL of Griess reagent (Sigma) for 20 min at room temperature, absorbance was measured using UV-visible spectrophotometer at 550 nm.

### Rat leukocyte preparation

We isolated rat leukocytes from whole blood. Whole blood was collected in a heparin tube and then centrifuged (3500 rpm; 10 min). After centrifuged, leukocyte pellet was collected and suspended in erythrocyte lysis buffer for 3 min. Erythrocyte lysis buffer was removed by centrifuge. Leukocyte pellet was washed with phosphate buffer saline (PBS) for three times. Leukocyte was collected and stored in -80 °C freezer.

### Preparing peritoneal macrophages

Four milliliters of 4 % thioglycollate solution was injected intraperitoneally, and the macrophages were harvested after 3 days in DMEM lavage medium. Ice-cold DMEM medium 10 mL was injected into the peritoneal cavity, gently mixed and removed. The collected cells were spun down and suspended in DMEM medium supplemented with 10 % heat-inactivated FBS, penicillin (100 U/mL), and streptomycin (100 U/mL). The peritoneal exudate cells contained 87-92 % macrophages and 6-9 % lymphocytes, as determined by morphological criteria. Macrophages were enriched from the total peritoneal exudates by adherence to plastic. Peritoneal exudate cells (2 x 10^6^ cells/mL) were seeded in 10-cm dishes. After the macrophages had been pre-cultured for 2 h, non-adherent cells were removed before treatment by washing them three times with ice-cold PBS.

### PPAR-gamma activation assay

Nuclear protein was obtained by using NE-PER nuclear and cytoplasmic extraction kit (Pierce, Austin, TX). PPAR-γ activation was detecting using PPAR-γ transcription factor assay kit (Cayman, Ann Arbor, MI).

### Cell viability assay?

Peritoneal macrophage viability was assayed by MTT assay. Macrophages (2 x 10^6^ cells/mL) were added new medium containing 0.5 mg/mL of MTT and cultured for 4 h. To the macrophage culture dimethyl sulfoxide was added and measured spectrophotometrically at 570 nm.

### Statistical analysis

The statistical analysis was done using the SPSS v. 11.0.1 software (SPSS Inc., IL, USA). All the data were presented as mean SD. One-way ANOVA followed by Tukey's Honestly Significant Difference (HSD) methods were used for comparisons between groups. *P* < 0.05 was considered to indicate statistical signiﬁcance.

## Results

### Sesamol maintained renal function and decreased cytokine levels in CLP induced AKI in rats

To evaluate the protective role of sesamol on CLP induced AKI and renal function, we analysed serum BUN and CRE. Serum BUN and CRE were significantly increased in CLP rats (Group II) relative to that of Sham rats (Group I). Sesamol significantly decreased serum BUN and CRE levels in CLP rats (Group III). To examine the effects of sesamol on the release of renal proinflammatory mediators, renal IL-1β and IL-6 were measured. Proinflammatory cytokines IL-1β and IL-6 levels were significantly increased in CLP rats (Group II) compared to Sham rats (Group I) and the sesamol alone treated rats (Group IV). However, sesamol (Group III) significantly decreased IL-1β and IL-6 levels in CLP rats (Table 1(Tab. 1)).

### Sesamol increased the MAP in LPS-induced hypotension 

**Figure 1:**
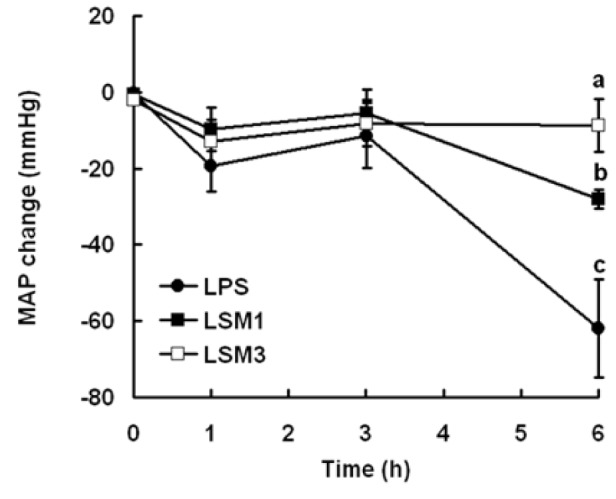
Effect of sesamol on LPS-induced hypotension. Rats were divided into three groups. LPS group, rats were treated with LPS (10 mg/kg, i.v.). LSM1- and LSM3-group rats were treated with sesamol (1 and 3 mg/kg, s.c., respectively) 1 h after LPS injection. Mean arterial pressure (MAP) was measured at 0, 1, 3, and 6 h. Data are means ± SD. Significant differences between measurement traits were analyzed using one-way ANOVA. Different letters indicate statistically significant (*P* < 0.05) differences between groups.

MAP was monitored to examine the effect of sesamol on LPS-induced hypotension. LPS induced MAP decrease in rats at 6 h. Sesamol (LSM1 and LSM3) significantly increased the MAP at 6 h compared with LPS-alone group (Figure 1(Fig. 1)). Therefore, we chose 3 mg/kg of sesamol at 6 h to examine the effect of sesamols anti-hypotension in the following experiments. 

### Sesamol modulated cytokine levels in LPS-induced hypotensive rats 

**Figure 2:**
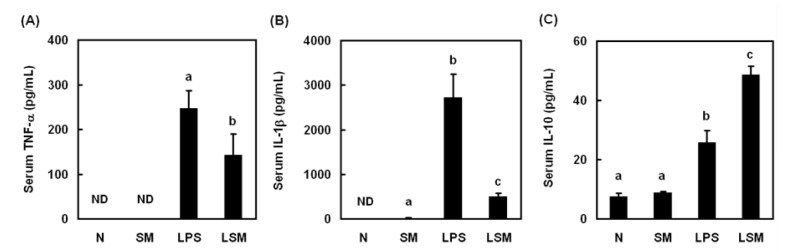
Effects of sesamol on pro-inflammatory and anti-inflammatory cytokines. Rats were divided into four groups: N group, rats were given saline only; SM group, rats were given sesamol (3 mg/kg, s.c.); LPS group, rats were given LPS (10 mg/kg, i.v.); LSM group, rats were given LPS then sesamol 1 h after LPS. Serum TNF-α, IL-1β, and IL-10 levels were assessed 6 h post-LPS injection. Data are means ± SD. Significant differences between measurement traits were analyzed using one-way ANOVA. Different letters indicate statistically significant (*P* < 0.05) differences between groups. (ND, not detected)

To investigate the role of cytokines in rats that had been treated with LPS to induce hypotension, serum TNF-α, IL-1β, and IL-10 levels were measured. LPS significantly increased the level of serum TNF-α (Figure 2A(Fig. 2)), IL-1β (Figure 2B(Fig. 2)), and IL-10 (Figure 2C(Fig. 2)) in LPS group. Sesamol inhibited serum pro-inflammatory cytokines TNF-α and IL-1β, and increased serum anti-inflammatory cytokine IL-10 in LSM group compared with LPS group.

### Sesamol had no effect on nitric oxide production 

**Figure 3:**
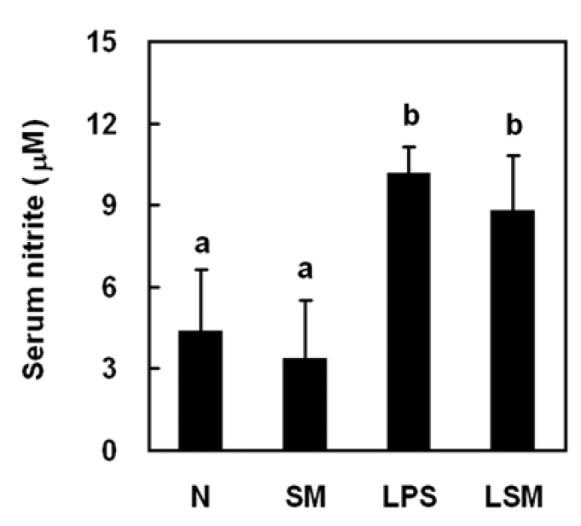
Effects of sesamol on serum nitrite (for treatment details, see legend for Figure 2). Serum nitrite level was assessed 6 h post-LPS injection. Data are means ± SD. Significant differences between measurement traits were analyzed using one-way ANOVA. Different letters indicate statistically significant (*P* < 0.05) differences between groups.

To comprehend the role of nitric oxide in sesamol-exerted blood pressure maintenance in LPS induced hypotension, serum nitrite level was measured. LPS significantly increased the serum nitrite level in LPS group compared with N and SM groups. Sesamol (LSM group) did not affect nitrite production compared with LPS group (Figure 3(Fig. 3)).

### Sesamol activated PPAR-gamma in rat leucocytes and LPS-treated macrophages 

**Figure 4:**
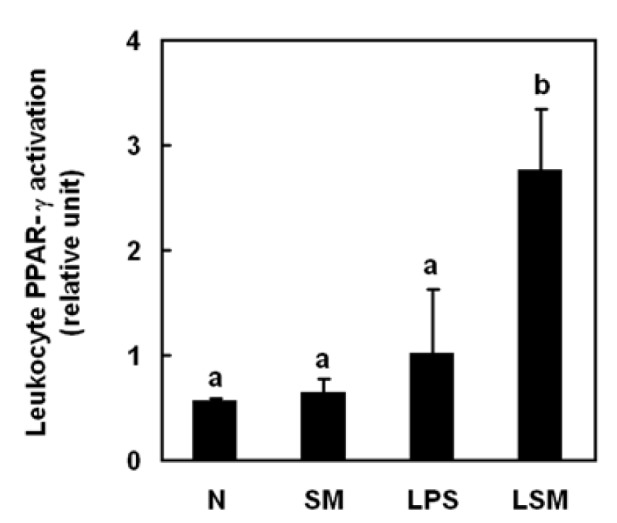
Effects of sesamol on peroxisome proliferator-activated receptor (PPAR)-γ activation in leukocytes (for treatment details, see legend for Figure 2). PPAR-γ activation in leukocytes was assessed 6 h post-LPS injection. Data are means ± SD. Significant differences between measurement traits were analyzed using one-way ANOVA. Different letters indicate statistically significant (*P* < 0.05) differences between groups.

To investigate the regulatory role of sesamol in LPS-induced SIR in rats, activation of PPAR-γ was measured in leucocytes. Sesamol (LSM group) significantly increased the level of PPAR-γ in leucocytes compared with LPS group (Figure 4(Fig. 4)). 

### Sesamol activated PPAR-gamma in LPS-treated primary macrophages 

**Figure 5:**
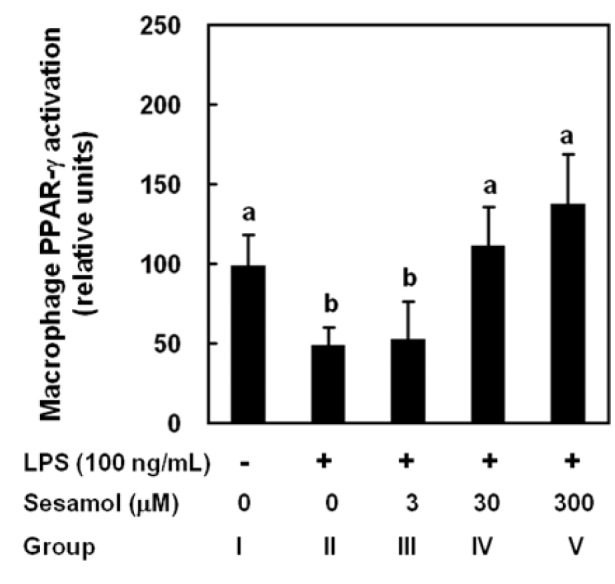
Effects of sesamol on PPAR-γ activation in LPS-treated primary peritoneal macrophages. Peritoneal macrophages were divided into five groups: Group I, cells were treated with PBS only; Group II, cells were treated with LPS (100 ng/mL); Group III-V, cells was treated with LPS (100 ng/mL) plus sesamol (3 νM, 30 νM, or 300 νM). PPAR-γ activation was measured 24 h after LPS treatment. Data are means ± SD. Significant differences between measurement traits were analyzed using one-way ANOVA. Different letters indicate statistically significant (*P* < 0.05) differences between groups.

To examine the immune regulation of sesamol in LPS-treated primary peritoneal macrophages, the activation of PPAR-γ was measured. LPS (Group II) significantly decreased PPAR-γ activation level in primary peritoneal macrophages compared with Group I. Sesamol (Group IV and Group V) significantly increased PPAR-γ activation in LPS-treated primary peritoneal macrophages (Figure 5(Fig. 5)) without affecting cell viability (data not shown).

## Discussion

In our previous studies, sesamol as a protective agent on pulmonary inflammatory response and lung injury, we injected sesamol (0.3, 1, and 3 mg/kg, respectively, s.c.) immediately after the LPS injection (10 mg/kg, i.p.) (Chu et al., 2011). We also studied the protective effect of one injection of sesamol (10 mg/ kg, s.c.) in down regulating nuclear factor-kappa B activation in LPS-induced inflammatory response and one intraperitoneal (i.p.) injection of LPS (5 mg/kg) (Chu et al., 2010[6]). In addition, we investigated the protective role of sesamol on LPS-induced oxidative stress and multiple organ injury, where injected sesamol (1 to 30 mg/kg, s.c.) just before an intraperitoneal (i.p.) LPS (5 mg/kg, i.p.) injection (Hsu et al., 2006b[14]). In contrast, the present investigation evaluated the post administration of sesamol and LPS (10 mg/kg) was given in i.v. infusion to check LPS-induced hypotension and the role of PPAR-γ in SIR rats.

Sesamol attenuated AKI in CLP-induced SIR and hypotension in LPS-induced SIR. Sesamol decreased AKI by reducing BUN and CRE and proinflammatory mediators in CLP model. In addition, it decreased TNF-α and IL-1β but increased IL-10, maintained MAP, increased leucocyte, macrophage PPAR-γ activation in LPS-induced SIR. We hypothesize that inhibiting TNF-α, IL-1β, and ΙL-6 and increasing IL-10 along with activation of leucocyte and macrophage PPAR-γ, thereby normalizing MAP in SIR-mediated AKI and hypotension.

Sesamol attenuated CLP-induced AKI by preventing the discharge of proinflammatory cytokines. In sepsis, cascade of events that include production of proinflammatory cytokines, free radicals by inflammatory cells that increase vascular permeability leading to AKI and hypotension (Yegenaga et al., 2004[34]). Sesamol maintained renal function and decreased proinflammatory mediators in CLP-induced SIR. For sepsis and systemic inflammation, LPS administration is used as an experimental model. Intravenous injection of LPS causes systemic effect that includes hypotension and increased cytokine release (Borge et al., 2009[3]). TNF-α and IL-1β are well known mediators of sepsis, released upon contact with LPS and the releases of proteases and arachidonic acid metabolites, which result in inflammation, vasodilation, and thrombosis (John et al., 2008[16]). Intravenous injection of LPS (3.5 mg/kg), TNF-α is significantly higher than control at all-time points compared with control at 30 min and peaked at 90 min and IL-6 is significantly elevated compared with control at 90 and 180 min (Borge et al., 2009[3]). LPS induced SIR in animals is characterized by increased production of proinflammatory cytokines, with the development of systemic hypotension (Liu et al., 1997[20]). TNF-α and IL-1β are the dominant pro-inflammatory mediators that trigger endothelium and inflammatory cells to amplify the SIR, which are involved in septic shock pathogenesis (Vallbracht-Israng et al., 2007[29]; Deswal et al., 2001[8]; Sundaresan and Sheagren, 1995[28]). Nitric oxide overproduction is a crucial factor in developing hypotension in septic shock (Ruggiero, 2008[25]; Wort and Evans, 1999[31]). Increase in the proinflammatory cytokines TNF-α and IL-1β is accompanied with marked decrease in MAP in LPS-induced murine septic shock model (Lehmann et al., 2008[19]; Gibot et al., 2006[11]). The increase of TNF-α and IL-1β might be due to pro-inflammatory mediators that trigger endothelium leading to vasodilation and thrombosis (John et al., 2008[16]) leading to alterations in MAP. Sesamol's intravenous intervention inhibited TNF-α and IL-1β without affecting nitrite level and increased IL-10 level and maintained MAP in LPS-induced hypotension and SIR. Although inhibiting nitric oxide production is associated with the anti-hypotensive effect of various vasopressors (Barrett et al., 2007[2]), sesamol did not inhibit nitric oxide overproduction in LPS-induced hypotension. In addition, IL-10 production effectively decreases proinflammatory cytokine production and balances the SIR (Woszczek et al., 2008[32]). Sesamol's direct inhibition of TNF- and IL-1β might alter the MAP; thereby it attenuated the hypotension (Lehmann et al., 2008[19]; Gibot et al., 2006[11]). Therefore, we hypothesize that sesamol increases IL-10, decreases the production of TNF- and IL-1β to balance the SIR against LPS-induced hypotension and endotoxemia. However, further studies are needed to prove this fact.

Sesamol-associated macrophage PPAR-γ activation may regulate LPS-induced hypotension and SIR. Activation of PPAR-γ plays a role in controlling the inflammatory response. In clinically relevant septic shock models activation of PPAR-γ with specific ligands significantly improved survival. PPAR-γ activation is beneficial and probably to be minor extend to inhibition of the release of several inflammatory mediators, *in vivo* in septic rodents and *in vitro* in activated macrophages and monocytes (Kaplan et al., 2010[17]; Collin et al., 2004[7]). In LPS-treated animals, the non-significant increase in the PPAR-γ activation might be the feedback mechanism at the end of 6 h. However, in *in vitro* PPAR-γ activation significantly decreased upon LPS stimulation. Activation of PPAR-γ inhibits inflammatory cytokine expression and directs the differentiation of immune cells towards anti-inflammatory phenotypes (Martin, 2009[21]). Further, PPAR-γ activation up-regulates IL-10 production (Kim et al., 2005[18]). Sesamol increased PPAR-γ activation in both rat leukocytes and LPS-treated primary macrophages. In addition, sesamol upregulated IL-10 production, which might be due to the activation of PPAR-γ. It is likely that PPAR-γ activation in macrophages has a role in sesamol-associated regulation of inflammatory cytokines in LPS-induced hypotension and SIR, at least partially.

The implication of the present investigation is that sesamol might be a useful candidate in treating end stage septic shock. Septic shock is life-threatening low blood pressure due to sepsis, a SIR syndrome caused by infection (Ruokonen et al., 2002[26]). The mortality of septic shock is estimated around 82 % (Salvo et al., 1995[27]). Maintaining blood pressure is a key for survival in septic patients. However, patients usually face the refractory hypotension despite adequate fluid resuscitation and high-dose conventional vasopressors including norepinephrine, dopamine, epinephrine, and vasopressin administration (Ruokonen et al., 2002[26]). Sesamol not only inhibits the binding between LPS and LPS-binding protein, but also competes with LPS-binding protein to bind to LPS (Hsu et al., 2009[13]). Sesamol might be effective after LPS is released into the blood. In addition, right and effective doses sesamol have no known side effects in rats (Hsu et al., 2009[13]). Therefore, developing an alternative approach such as sesamol to manage hypotension in patients with septic shock would be important. However, more investigation is needed. Thus, we conclude that sesamol attenuates LPS-induced hypotension by regulating the circulating inflammatory response through PPAR-γ-associated mechanism in rats.

## Acknowledgements

This research was supported by NSC 99-2314-B-006-031-MY3 from the National Science Council, Taiwan.

## Conflict of interest

The authors declare that there are no conflicts of interest.

## Figures and Tables

**Table 1:**
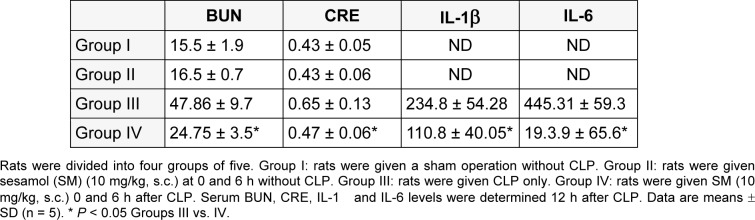
The effect of sesamol on BUN, CRE, IL-1β, and IL-6 levels in CLP-treated rats
